# Does geography influence the treatment and outcomes of colorectal cancer? A population-based analysis

**DOI:** 10.1186/1477-7819-11-140

**Published:** 2013-06-17

**Authors:** Ramzi M Helewa, Donna Turner, Debrah Wirtzfeld, Jason Park, David Hochman, Piotr Czaykowski, Harminder Singh, Emma Shu, Lin Xue, Andrew McKay

**Affiliations:** 1 Department of Surgery, University of Manitoba, AE101–820 Sherbrook Street, Winnipeg, MB R3A 1R9, Canada; 2 CancerCare Manitoba, 675 McDermot Avenue, Winnipeg, MB R3E 0V9, Canada; 3 Community Health Sciences, S113 Medical Services Building, University of Manitoba, 750 Bannatyne Avenue, Winnipeg, MB R3E 0W3, Canada; 4 Department of Internal Medicine, GC425 Health Sciences Centre, University of Manitoba, 820 Sherbrook Street, Winnipeg, MB R3T 2N2, Canada; 5 Department of Medical Oncology/Hematology, GF336A Health Sciences Centre, University of Manitoba, 820 Sherbrook Street, Winnipeg, MB R3A 1R9, Canada; 6 Departments of Surgery and Community Health Sciences, University of Manitoba, GF441–820 Sherbrook Street, Winnipeg, MB R3A 1R9, Canada

**Keywords:** Colorectal cancer, Quality of care, Geography, Manitoba

## Abstract

**Background:**

The Canadian province of Manitoba covers a large geographical area but only has one major urban center, Winnipeg. We sought to determine if regional differences existed in the quality of colorectal cancer care in a publicly funded health care system.

**Methods:**

This was a population-based historical cohort analysis of the treatment and outcomes of Manitobans diagnosed with colorectal cancer between 2004 and 2006. Administrative databases were utilized to assess quality of care using published quality indicators.

**Results:**

A total of 2,086 patients were diagnosed with stage I to IV colorectal cancer and 42.2% lived outside of Winnipeg. Patients from North Manitoba had a lower odds of undergoing major surgery after controlling for other confounders (odds ratio (OR): 0.48, 95% confidence interval (CI): 0.26 to 0.90). No geographic differences existed in the quality measures of 30-day operative mortality, consultations with oncologists, surveillance colonoscopy, and 5-year survival. However, there was a trend towards lower survival in North Manitoba.

**Conclusion:**

We found minimal differences by geography. However, overall compliance with quality measures is low and there are concerning trends in North Manitoba. This study is one of the few to evaluate population-based benchmarks for colorectal cancer therapy in Canada.

## Background

Canadian health care is publicly administered and universal for all insured residents [1]. However, authors have raised concern over suboptimal or unequal access and quality of health care among certain Canadian populations [2,3]. In Manitoba, colorectal cancer (CRC) is the third most common malignancy and poses a major public health issue [4]. Manitoba covers a large geographic area with 56.5% of the population living in the major urban center of Winnipeg [5,6]. Most cancer patients in Manitoba are referred to the provincially mandated agency, CancerCare Manitoba (CCMB), for consideration of neoadjuvant and adjuvant therapies, such as chemotherapy and radiation therapy. Two tertiary locations are found in Winnipeg, while other non-tertiary affiliated sites, providing chemotherapy, are found within both Winnipeg and several communities in rural Manitoba [7]. Although surgical facilities are present in both Winnipeg and rural Manitoba, the only radiation therapy unit, during the study period, was at the tertiary location of CCMB in Winnipeg. For a large proportion of Manitobans, access to specialized medical or surgical care requires travelling great distances, personal expense, and inconvenience as medical specialists are less frequently found in remote and rural environments.

Since the quality of treatment for CRC has recently become an important area for research and quality improvement initiatives [8-10], authors have developed a set of quality indicators for CRC treatment [11]. Quality measures permit identification of suboptimal practice patterns and allow opportunities for improvement [12]. Utilization and application of quality indicators permits benchmarking between institutions.

To date, there has been no formal analysis of the quality of CRC therapy in Manitoba using published quality measures. We sought to assess whether geographic differences existed in CRC quality of care. This study has important implications for Canada and other jurisdictions around the world that face similar challenges in offering specialized health care to rural populations over great distances.

## Methods

The Health Research Ethics Board (HREB) at the University of Manitoba, MB, Canada, approved this study. A population-based historical cohort analysis of all patients diagnosed with adenocarcinoma of the colon or rectum between 1 January 2004 and 31 December 2006 was undertaken to examine the presentation, treatments, and outcomes of CRC in Manitoba. Patients were identified using the population-based Manitoba Cancer Registry (MCR), maintained by CCMB. Information regarding all Manitobans diagnosed with a malignancy is collected by the MCR as cancer reporting is mandatory by Manitoba law [13]. The MCR was used to identify patients based on the International Statistical Classification of Diseases and Related Health Problems, 10th Revision (ICD-10). Patients who were diagnosed with CRC at the same date as death, through autopsy or radiographic findings, were excluded (n = 3).

From the MCR, demographic and tumor-specific information, such as collaborative American Joint Committee on Cancer (AJCC) stage, were extracted. Patient-specific data from the MCR was linked to information in the Medical Claims (physician billing) database and the Hospital Discharge Abstracts database. Although health care utilization can be followed longitudinally, patient information was linked using encrypted personal health information numbers to maintain patient confidentiality. These databases are maintained by Manitoba Health, the agency responsible for providing health care to virtually all Manitobans, and contain patient-specific information about health care system contacts.

For treatment information from the MCR, the International Classification of Diseases, 9th Revision, Clinical Modification (ICD-9-CM) and the Canadian Classification of Health Interventions (CCHI) coding were used [14]. All treatment information was ascertained from within 1 year of diagnosis of CRC. Index surgical procedures, identified from the MCR, were classified in a hierarchical pattern into: major surgery, local resections, polypectomy, and none. The level of agreement for surgical procedures between the MCR and administrative records from Manitoba Health was determined using Fleiss’ kappa.

The quality of CRC care was assessed using previously published quality indicators, developed by a multidisciplinary panel using a 3-step modified Delphi approach [11]. A subset of these indicators was chosen on the ability to accrue data from administrative databases.

### Perioperative total colonic examination

For patients who underwent a major surgical resection, the proportion who had a colonoscopy alone or a barium/contrast enema in conjunction with sigmoidoscopy 3 months prior or within 6 months after major surgery was determined [11,15].

### Anastomotic leak/early reoperation rate

No diagnostic code currently exists for anastomotic leaks after surgery. To estimate this rate, surrogate markers were utilized. For patients who underwent a major surgery with an anastomosis that had one of the following procedures during the same hospital stay, an anastomotic leak was assumed: percutaneous or operative drainage of an intra-abdominal abscess, laparotomy or laparoscopy post major surgery, or ostomy formation post major surgery. Although a laparotomy or laparoscopy in the postoperative period may have been indicated for other complications, such as bleeding or obstruction, these were included as early reoperation also represents a quality measure.

### Extent of lymphadenectomy

Using the MCR, we assessed the median number of lymph nodes analyzed for patients undergoing a major surgical resection and the proportion of patients in which greater than 12 lymph nodes were examined [16-19].

### Medical and radiation oncology consultations for patients with rectal cancer

The proportion of all patients with stage I to IV, as well as stage II and III, rectal cancer who had been seen by a medical and/or a radiation oncologist 3 months prior to major surgery was determined from the MCR. In addition, the proportion of patients with stage II or III disease seen within 8 weeks, as well as within 16 weeks, of major surgery was reported.

### Medical oncology consultations for patients with colon cancer

The proportion of patients with stage III colon cancer who had been seen by a medical oncologist within 8 weeks and 16 weeks of surgery was determined.

### Thirty-day mortality

The 30-day mortality rate after colon or rectal cancer major surgery was determined.

### Postoperative endoscopic surveillance

For patients who underwent a major surgery, we determined the proportion of patients, who were alive, and who underwent a surveillance colonoscopy within 12 months and 14 months after the surgery date. The 1-year post surgery surveillance colonoscopy is recommended by the National Comprehensive Cancer Network (NCCN) [20,21] and has been prioritized to be a quality measure by an expert multidisciplinary panel [11].

### Five-year overall survival rate

The 5-year overall absolute Kaplan-Meier survival estimates from date of diagnosis to death, was determined using mortality data obtained up to 30 April 2011.

Geographical comparisons were made between Winnipeg and rural Manitoba, as well as between groupings of Manitoba’s regional health authorities (RHAs): Winnipeg, North Manitoba, South Manitoba, and Middle Manitoba. Manitoba’s RHAs are regional governances whose responsibility is to administer and deliver health services to specified geographic regions of the province [22].

Standard descriptive statistics and treatment frequency information were reported. Treatment information from the MCR was compared to the information taken from the Hospital Discharge Abstracts to check the validity of the data. Conventional stepwise multivariate logistic regression was used to determine variables associated with major surgery.

Survival was analyzed using Kaplan-Meier survival analysis. The Cox proportional hazards regression model was used to determine variables associated with overall survival. Significance was set at α = 0.05. SAS statistical software, versions 9.1 and 9.2 (SAS Institute Inc, Cary, NC, USA), was used for data management and statistical analyses.

## Results

Between 2004 and 2006, 2,086 patients were diagnosed with stage I to IV adenocarcinoma-based CRC. Table 1 lists patient demographic information and comparisons by RHA group. There was relatively equal distribution of patients diagnosed between 2004 and 2006. A higher proportion of younger patients and a trend towards more advanced stage at presentation were noted for patients from North Manitoba.

**Table 1:** Demographics and tumor characteristics of patients diagnosed with stage I to IV CRC

		**RHA group**				
**Variable**	**Overall (N = 2,086)**	**Winnipeg (n = 1,206)**	**North Manitoba (n = 65)**	**South Manitoba (n = 496)**	**Middle Manitoba (n = 319)**	***P*value**
Mean age in years (range)	70 ± 13.00	70 ± 13.21	65 ± 13.06	70 ± 13.08	71 ± 11.80	0.004
	(20 to 103)	(25 to 103)	(31 to 92)	(20 to 94)	(39 to 94)	
Age group (n (%))						
>70 years	1,182 (56.66)	695 (57.63)	25 (38.46)	277 (55.85)	185 (57.99)	0.022
<70 years	904 (43.34)	511 (42.37)	40 (61.54)	219 (44.15)	134 (42.01)	
Age quartiles^a^ (n (%))						
Quartile 1	542 (25.98)	324 (26.87)	27 (41.54)	129 (26.01)	62 (19.44)	0.004
Quartile 2	522 (25.02)	282 (23.38)	17 (26.15)	120 (24.19)	103 (32.29)	
Quartile 3	530 (25.41)	312 (25.87)	12 (18.46)	128 (25.81)	78 (24.45)	
Quartile 4	492 (23.59)	288 (23.88)	9 (13.85)	119 (23.99)	76 (23.82)	
Gender (n (%))						
Female	963 (46.16)	576 (47.76)	29 (44.62)	223 (44.96)	135 (42.32)	0.323
Male	1,123 (53.84)	630 (52.24)	36 (55.38)	273 (55.04)	184 (57.68)	
Diagnosis year (n (%))						
2004	706 (33.84)	413 (34.25)	20 (30.77)	164 (33.06)	109 (34.17)	0.932
2005	671 (32.17)	389 (32.26)	25 (38.46)	156 (31.45)	101 (31.66)	
2006	709 (33.99)	404 (33.50)	20 (30.77)	176 (35.48)	109 (34.17)	
Site (n (%))						
Colon	1,376 (65.96)	788 (65.34)	44 (67.69)	336 (67.74)	208 (65.20)	0.486
Rectosigmoid	202 (9.68)	116 (9.62)	7 (10.77)	54 (10.89)	25 (7.84)	
Rectum	508 (24.35)	302 (25.04)	14 (21.54)	106 (21.37)	86 (26.96)	
AJCC stage (n (%))						
I	403 (19.32)	226 (18.74)	7 (10.77)	114 (22.98)	56 (17.55)	0.069^c^
II	575 (27.56)	334 (27.69)	19 (29.23)	139 (28.02)	83 (26.02)	
III	610 (29.24)	364 (30.18)	15 (23.08)	134 (27.02)	97 (30.41)	
IV	439 (21.05)	255 (21.14)	20 (30.77)	95 (19.15)	69 (21.63)	
Unknown/NA^b^ (n (%))	59 (2.83)	27 (2.24)	4 (6.15)	14 (2.82)	14 (4.39)	

^a^Quartile 1: age 20 years to 61 years, quartile 2: age greater than 61 years to 72 years, quartile 3: age greater than 72 years to 80 years, and quartile 4: age greater than 80 years to 103 years; ^b^one or more elements of the AJCC stage is missing or staging scheme is not applicable; ^c^usage of Monte-Carlo estimation of exact *P* value instead of direct *P* value computations, since the sample size is large and some cell values are less than five. *AJCC* American Joint Committee on Cancer, *CRC* colorectal cancer, *NA* not applicable, *RHA* regional health authority.

Of all patients diagnosed with CRC, 78.04% (n = 1,628) underwent a major surgery. Fifty-eight patients (2.78%) underwent a local resection, while 70 patients (3.35%) underwent a polypectomy alone. A total of 330 patients (15.82%) did not have any surgical intervention (neither major surgery, local resection, nor polypectomy). Predictors of major surgery were analyzed using a stepwise multivariate logistic regression (Table 2). Patients diagnosed with rectal cancer, stage IV disease, and those from North Manitoba were at a lower odds of undergoing major surgery (odds ratio (OR): 0.48, 95% confidence interval (CI): 0.26 to 0.90, *P* = 0.037). Fleiss’ kappa level of agreement between the MCR and Hospital Discharge Abstracts was 0.788 for overall surgical categories (major surgery: 0.821, local resection: 0.615, polypectomy: 0.606, and none: 0.821).

**Table 2:** Stepwise multivariate logistic regression for occurrence of a major surgical resection using RHA group

**Variable**	**Adjusted OR**^a^	**95% CI**	***P*value**
Site			<0.0001
Colon	1		
Rectosigmoid	0.76	0.51 to 1.14	
Rectum	0.53	0.40 to 0.70	
AJCC stage			<0.0001
I	1		
II	4.11	2.83 to 5.97	
III	5.97	3.87 to 9.20	
IV	0.34	0.24 to 0.48	
Unknown/NA^b^	0.09	0.04 to 0.19	
Income quintile^c^			0.045
Quintile1	1		
Quintile 2	1.04	0.73 to 1.48	
Quintile 3	1.37	0.94 to 1.98	
Quintile 4	1.41	0.96 to 2.07	
Quintile 5	1.26	0.85 to 1.86	
NF	0.32	0.11 to 0.93	
CCI group			0.045
CCI count <1	1		
CCI count = 1	1.39	1.00 to 1.93	
CCI count >1	0.96	0.68 to 1.35	
RHA group			0.037
Winnipeg	1		
North Manitoba	0.48	0.26 to 0.90	
South Manitoba	1.25	0.93 to 1.69	
Middle Manitoba	1.11	0.78 to 1.57	

Potential covariates in the model included: age group (<70 years, >70 years), gender, site (colon, rectosigmoid, rectum), AJCC stage, income quintile, Charlson comorbidity index (CCI) group, and RHA group. Age and gender were not significant in the stepwise analysis.

^a^The higher the odds ratio, the higher the odds of undergoing major surgery; ^b^one or more elements of the AJCC stage is missing or staging scheme is NA; ^c^quintile 1: poorest, quintile 5: richest, NF: patients for whom income quintile information was not found, including patients living in institutionalized facilities, such as personal care homes, mental health institutes, prisons, or offices of the public trustee. *AJCC* American Joint Committee on Cancer, *CCI* Charlson comorbidity index, *CI* confidence interval, *NA* not applicable, *NF* not formatted, *OR* odds ratio, *RHA* regional health authority.

Quality measures and geographic variations are shown in Table 3. The postoperative (30-day) mortality rate was 3.8%. Overall, 75.6% of patients (1,230/1,628) had perioperative total colonic examination. There were 217 patients (13.3%) that did not have any colonic/endoscopic imaging before or after surgery. The extent of lymphadenectomy could be determined for 96.5% of patients undergoing resection.

**Table 3:** Quality measures for patients diagnosed with CRC between 2004 and 2006, and comparisons by geography

		**Geographic variation**			**RHA group**				
**Quality measure**	**Overall (n/N)**	**Winnipeg (n/N)**	**Other (n/N)**	***P*value**	**Winnipeg**	**North Manitoba**	**South Manitoba**	**Middle Manitoba**	***P*value**
					**(n/N)**	**(n/N)**	**(n/N)**	**(n/N)**	
Total colonic examination	75.55%	75.08%	76.20%	0.604	75.08%	65.00%	74.00%	81.53%	0.049
	(1,230/1,628)	(705/939)	(525/689)		(705/939)	(26/40)	(296/400)	(203/249)	
Anastomotic leak/reoperation rate	1.72%	2.17%	1.13%	0.168	2.17%	2.94%	1.34%	0.51%	0.355^a^
	(21/1,220)	(15/691)	(6/529)		(15/691)	(1/34)	(4/298)	(1/197)	
Extent of lymphadenectomy									
>12 lymph nodes	68.75%	69.07%	68.31%	0.749	69.07%	58.97%	72.42%	63.22%	0.054
	(1,080/1,571)	(623/902)	(457/669)		(623/902)	(23/39)	(281/388)		
Positive lymph node status	45.47%	45.76%	45.09%	0.790	45.76%	48.72%	43.48%	47.11%	0.781
	(719/1,581)	(416/909)	(303/672)		(416/909)	(19/39)	(170/391)	(114/242)	
Medical oncology for rectal cancer									
Stage I to IV preoperative consultation	13.15%	12.90%	13.51%	0.866	12.90%	28.57%	13.51%	11.94%	0.668
	(48/365)	(28/217)	(20/148)		(28/217)	(2/7)	(10/74)	(8/67)	
Stage II/III preoperative consultation	16.25%	14.48%	18.95%	0.359	14.48%	40.00%	22.73%	13.04%	0.250
	(39/240)	(21/145)	(18/95)		(21/145)	(2/5)	(10/44)	(6/46)	
Stage II/III seen within 8 weeks of major surgery	36.25%	37.24%	34.74%	0.693	37.24%	40.00%	43.18%	26.09%	0.382
	(87/240)	(54/145)	(33/95)		(54/145)	(2/5)	(19/44)	(12/46)	
Stage II/III seen within 16 weeks of major surgery	63.33%	63.45%	63.16%	0.964	63.45%	60.00%	77.27%	50.00%	0.065
	(152/240)	(92/145)	(60/95)		(92/145)	(3/5)	(34/44)	(23/46)	
Radiation oncology for rectal cancer									
Stage I to IV preoperative consultation	17.81%	18.43%	16.89%	0.706	18.43%	14.29%	18.92%	14.93%	0.906
	(65/365)	(40/217)	(25/148)		(40/217)	(1/7)	(14/74)	(10/67)	
Stage II/III preoperative consultation	22.50%	23.45%	21.05%	0.664	23.45%	20.00%	27.27%	15.22%	0.559
	(54/240)	(34/145)	(20/95)		(34/145)	(1/5)	(12/44)	(7/46)	
Stage II/III seen within 8 weeks of major surgery	31.25%	33.79%	27.37%	0.294	33.79%	0%	27.27%	30.43%	0.384
	(75/240)	(49/145)	(26/95)		(49/145)	(0/5)	(12/44)	(14/46)	
Stage II/III seen within 16 weeks of major surgery	67.08%	68.28%	65.26%	0.627	68.28%	40.0%	77.27%	56.52%	0.105
	(161/240)	(99/145)	(62/95)		(99/145)	(2/5)	(34/44)	(26/46)	
Stage III colon cancer seen within 8 weeks of surgery	27.27%	27.35%	27.15%	0.966	27.35%	55.56%	28.92%	20.34%	0.160
	(102/374)	(61/223)	(41/151)		(61/223)	(5/9)	(24/83)	(12/59)	
Stage III colon cancer seen within 16 weeks of surgery	50.80%	47.09%	56.29%	0.081	47.09%	77.78%	63.86%	42.37%	0.011
	(190/374)	(105/223)	(85/151)		(105/223)	(7/9)	(53/83)	(25/59)	
30-day mortality	3.75%	4.05%	3.34%	0.506^^^	4.05%	2.5%	4.00%	2.41%	0.643^^^
	(61/1,628)	(38/939)	(23/689)		(38/939)	(1/40)	(16/400)	(6/249)	
Surveillance colonoscopy within 12 months of surgery	29.62%	30.06%	29.02%	0.674	30.06%	25.00%	31.33%	26.05%	0.535
	(410/1,384)	(242/805)	(168/579)		(242/805)	(8/32)	(104/332)	(56/215)	
Surveillance colonoscopy within 14 months of surgery	45.37%	47.22%	42.81%	0.106	47.22%	32.26%	45.12%	40.76%	0.167
	(618/1,362)	(374/792)	(244/570)		(374/792)	(10/31)	(148/328)	(86/211)	

^a^Usage of Monte-Carlo estimation of exact *P* value instead of direct *P* value computations, since the sample size is large and some cell values are less than five; ^death within 30 days compared to death after 30 days and patients who were still alive. *CRC* colorectal cancer, *RHA* regional health authority.

**Figure 1:**
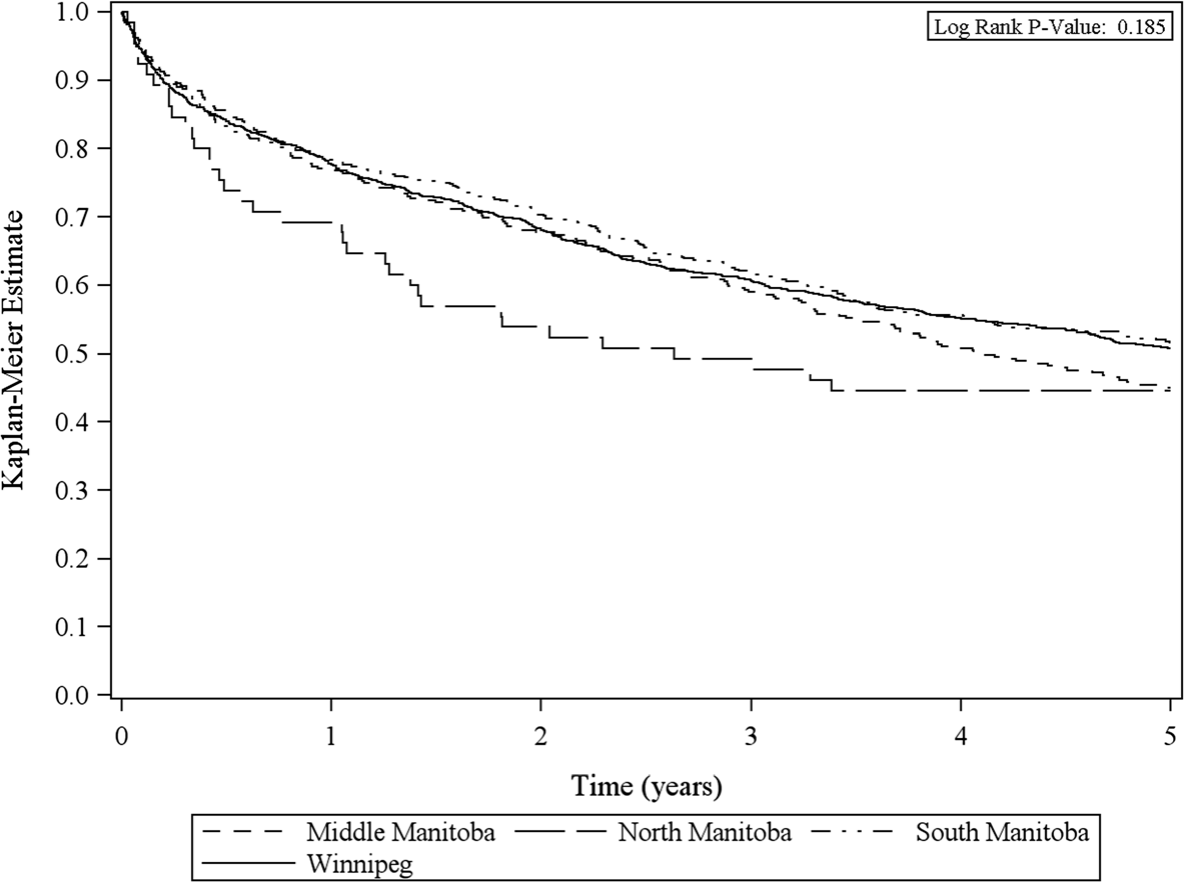
Kaplan-Meier 5-year survival estimate by RHA group.

The 5-year overall survival for all patients diagnosed with CRC between 2004 and 2006 was 49.9%. There was no significant difference based on geography in Manitoba, although a trend towards decreased survival for patients in North Manitoba was observed (Figure 1). The multivariate Cox proportional hazards model analysis for 5-year overall survival is shown in Table 4.

**Table 4:** **The 5**-**year overall survival Cox proportional multivariate hazards model**

**Variable**	**Event (n (%))**	**Adjusted HR**^**a**^	**95%CI**	***P*value**
Age quartile^b^				<0.0001
Quartile 1	210 (38.75)	1		
Quartile 2	234 (44.83)	1.21	1.00 to 1.46	
Quartile 3	266 (50.19)	1.59	1.31 to 1.93	
Quartile 4	328 (66.67)	2.07	1.69 to 2.53	
Gender				0.007
Female	470 (48.81)	1		
Male	568 (50.58)	1.19	1.05 to 1.35	
Site				0.755
Colon	707 (51.38)	1		
Rectosigmoid	103 (50.99)	1.06	0.85 to 1.31	
Rectum	228 (44.88)	0.96	0.81 to 1.15	
AJCC stage				<0.0001
I	111 (27.54)	1		
II	188 (32.70)	1.44	1.12 to 1.86	
III	279 (45.74)	2.22	1.71 to 2.87	
IV	412 (93.85)	7.67	5.88 to 10.00	
Unknown/NA^c^	48 (81.36)	3.08	2.14 to 4.45	
Income quintile^d^				0.004
Quintile 1	271 (58.66)	1		
Quintile 2	240 (52.86)	1.07	0.90 to 1.28	
Quintile 3	209 (47.83)	0.95	0.79 to 1.14	
Quintile 4	159 (42.51)	0.92	0.75 to 1.13	
Quintile 5	137 (40.90)	0.77	0.63 to 0.95	
NF	22 (91.67)	1.78	1.14 to 2.78	
CCI group				<0.0001
CCI count <1	235 (28.08)	1		
CCI count = 1	448 (58.56)	2.03	1.70 to 2.42	
CCI count >1	355 (73.35)	2.79	2.33 to 3.36	
RHA group				0.314
Winnipeg	589 (48.84)	1		
North Manitoba	36 (55.38)	0.98	0.69 to 1.39	
South Manitoba	239 (48.19)	1.16	0.99 to 1.35	
Middle Manitoba	174 (54.55)	1.04	0.87 to 1.23	
Surgery				<0.0001
Major surgery	694 (42.63)	0.54	0.40 to 0.73	
Local resection	14 (24.14)	0.33	0.18 to 0.58	
Polypectomy	26 (37.14)	0.63	0.40 to 0.97	
None	304 (92.12)	1		
Chemotherapy				<0.0001
No	677 (50.52)	1		
Yes	361 (48.39)	0.59	0.50 to 0.69	
Radiation therapy				0.126
No	916 (51.29)	1		
Yes	122 (40.67)	0.84	0.67 to 1.05	
Lymph nodes removed				<0.0001
0 to 12	249 (47.43)	1		
>12	440 (39.43)	0.76	0.65 to 0.89	
Unknown/not examined^e^	349 (78.43)	1.43	1.06 to 1.94	

All variables listed are included in the full model. ^a^HR >1 relates to higher mortality; ^b^quartile 1: age 20 years to 61 years, quartile 2: age greater than 61 years to 72 years, quartile 3: age greater than 72 years to 80 years, and quartile 4: age greater than 80 years to 103 years; ^c^one or more elements of the AJCC stage is missing or staging scheme is NA; ^d^quintile 1: poorest, quintile 5: richest; ^e^unknown number or no lymph nodes examined. *AJCC* American Joint Committee on Cancer, *CCI* Charlson comorbidity index, *CI* confidence interval, *HR* hazards ratio, *NA* not applicable, *NF* not formatted, *RHA* regional health authority.

## Discussion

In Manitoba, between 2004 and 2006, there was minimal geographic variation of certain quality measures for CRC. However, overall compliance with many quality measures appears low. Addressing these issues is paramount in order to provide all Manitobans with coordinated, high quality care.

It was hypothesized that differences in access to cancer care could manifest with rural patients presenting with higher stage disease. This hypothesis was not confirmed. However, there was a trend towards a higher stage in patients from North Manitoba. This observation is of concern and requires further research.

Over 78% of patients underwent a major surgery, similar to findings in Ontario, Canada [23]. After controlling for stage and other confounders, living in North Manitoba at the time of diagnosis was associated with a lower chance of undergoing surgery. Both system- and patient-related factors could explain this disparity. Patient-related factors in health decision making may be numerous, including age, gender, education, emotional support, and physician trust [24]. Anxiety, personal beliefs, or aversion to postoperative complications may influence patients to not seek or receive therapy [25,26]. Humber and Dickinson examined rural patients’ experiences of accessing surgery in British Columbia, Canada [27]. They determined that rural patients prefer individualized care in familiar environments. Transportation and financial barriers were noted to be detrimental factors. Rural patients may be viewed as a culture with their own health determinants and challenges in accessing health care [28]. The psychological effects of a cancer diagnosis compounded with the need to travel to new environments in order to access specialist care can be profound and may deter patients from seeking therapies. System-related factors, such as barriers to timely referral to surgeons, or patients being offered different treatments may also play a role.

Perioperative total colonic examination is important, since the reported incidence of synchronous colonic lesions ranges from 2.12% to 8.1% [29-33]. In Manitoba, 75.6% of patients met this quality measure, similar to a study from Nova Scotia, Canada [10]. Rates of total colonic examination were lower in North Manitoba. It can be hypothesized that this may relate to endoscopy access issues. Also, patients may present emergently and be unable to undergo full preoperative evaluation, and subsequently not undergo evaluation postoperatively. It is concerning that in our analysis, 13.3% of patients did not have any colonic investigations. The underlying reasons are unknown, but present an important area for further research. Additionally, our reported rate of surveillance colonoscopy within 1 year of surgery of 29.62% is lower than that reported by other authors [16].

Adequate lymphadenectomy is a well-established quality measure of colorectal cancer care for its vital role in appropriate staging [17,34]. Lymph node status is a strong predictor of survival outcomes in non-metastatic CRC and implies the need for adjuvant therapy [17,35-38]. Patients who had greater than 12 lymph nodes examined did, in fact, have a lower risk of mortality in this analysis. Adequate lymphadenectomy may provide both a direct therapeutic benefit, and improve staging accuracy and prognosis [39]. A total of 68.8% of patients had adequate nodal evaluation, as reflected by more than 12 lymph nodes examined in pathology specimens. This rate is higher than that reported by others [10,16-18], yet still presents an opportunity for improvement.

Timely and appropriate assessment of patients diagnosed with rectal cancer by medical and radiation oncology, either preoperatively or within 8 weeks of surgery, is an important quality measure [11]. Previous studies have been unable to determine rates of consultation, and instead reported the proportion of patients that received radiation or chemotherapy [16]. Reporting rates of consultation presents a more accurate measure of quality of care. The rates of assessment by medical and radiation oncology within 8 weeks for patients with rectal cancer are quite low. Others have reported higher rates [10,40]. When extending the timeframe to 16 weeks, the rate of consultation with medical and radiation oncology nearly doubled. This suggests that referrals are being made appropriately but system limitations may account for undue delays in patients being seen. Referral delays, wait lists, and pathology reporting delays may contribute to lower than expected adherence with the quality measures, and could explain the increased rate of patients seen within 16 weeks. During the study period, Manitoba’s radiation therapy services were only available at a single center in Winnipeg, necessitating travel for rural patients. In addition, the proportion of patients with rectal cancer seen preoperatively by radiation oncology was low, possibly because the timeframe of this study predated the general trend from adjuvant to neoadjuvant chemoradiation treatment [41].

For patients diagnosed between 2004 and 2006, Manitoba’s population-based 5-year overall survival was 49.9%. Initially, this appears to be lower than previously reported [16,42]. However, comparisons must be made cautiously. Vergarara-Fernandez *et al*. reported a 5-year overall survival rate of 75% at a single, high volume tertiary center [16]. Population-based absolute survival rates are presented in our study. Most notably, no geographic differences in 5-year survival were demonstrated. However, though not statistically significant, a trend towards a lower 5-year overall Kaplan-Meier survival rate for patients from North Manitoba was noted. This trend highlights an important area for future research.

This study is limited by the retrospective collection of administrative data, which may contain incomplete records and coding errors [43-45]. However, the MCR has been demonstrated to be among the highest quality administrative cancer databases [46]. Based on the study design, patients’ preferences could not be accounted for. Additionally, we were unable to report relative survival rates to control for underlying mortality in each RHA group. This limits the ability to draw comparisons to other population-based survival analyses. Furthermore, we were unable to account for racial differences in outcomes in this analysis. However, this is the first Manitoban study to provide comprehensive CRC quality of care benchmarks, which can be used for future temporal and interprovincial comparisons. Additionally, there was a high level of agreement between the MCR and the administrative databases maintained by Manitoba Health for surgical treatment information. This finding may allow for future studies to use the MCR for surgical cancer treatment information.

We have identified concerning findings in North Manitoba, such as the lower odds of receiving major surgery, while accounting for stage and other confounders, and lower rates of total colonic examination. The trend towards lower survival and higher stage in North Manitoba is very concerning. Given that only 65 patients were from North Manitoba, this study suffered from lack of power when analyzing this subset of the population further. The MCR only began collecting detailed TNM staging information in 2004, preventing the addition of earlier years to the study data. Future analyses, including later time periods, beyond 2006, will be undertaken to better delineate the findings for patients in North Manitoba. However, these findings are important and should be shared with other jurisdictions that share similar geographical challenges in providing high quality care for CRC and other medical problems. Focus should be placed on addressing access, surgical issues, such as patients’ preferences, in addition to system-based barriers for patients living in locations remote from major urban centers. Further research on rural patients’ perspectives of surgery is warranted.

## Conclusion

Although minimal geographic differences in quality measures were seen, overall adherence was less than ideal. Further research is necessary to better delineate the reasons for this. This research is important in its implication for Manitoba’s health care system, as well as for the rest of Canada, and other areas of the world that might face similar challenges in providing high quality cancer care to rural patients over significant distances.

## Abbreviations

AJCC: American joint committee on cancer; CCHI: Canadian classification of health interventions; CCI: Charlson comorbidity index; CCMB: CancerCare Manitoba; CI: Confidence interval; CRC: Colorectal cancer; HR: Hazards ratio; HREB: Health research ethics board; ICD-9-CM: International classification of diseases 9th revision, clinical modification; ICD-10: International statistical classification of diseases and related health problems 10th revision; MCR: Manitoba cancer registry; NA: Not applicable; NCCN: National comprehensive cancer network; NF: Not formatted; OR: Odds ratio; RHA: Regional health authority.

## Competing interests

The authors declare that they have no competing interests.

## Authors’ contributions

RMH and AM made substantial contributions to study conception, design, acquisition, analysis and interpretation of data, as well as drafting and critically revising the manuscript. DT made substantial contributions to study conception, design, analysis and interpretation of data, as well as drafting and revising the manuscript. DW, JP, DH, PC, and HS made substantial contributions to study conception, design, analysis and interpretation of data, as well as critically revising the manuscript. ES and LX made substantial contributions to acquisition, analysis and interpretation of data, as well as critically revising the manuscript. All authors read and approved the final manuscript.

## Synopsis

Minimal geographic differences exist in most colorectal cancer quality measures in Manitoba, Canada. However, overall compliance rates are low, and we have identified trends towards higher stage at presentation and lower rates of surgery in North Manitoba.

Helewa RM, Turner D, Wirtzfeld D, Park J, Shu E, Xue L, McKay A. **Does geography influence the treatment and outcomes of colorectal cancer in the province of manitoba?** Presented at *Society of Surgical Oncology, 65th Annual Cancer Symposium*. Rosemont: Society of Surgical Oncology; March 2012.
